# Effects of Various Disinfection Methods on the Material Properties of Silicone Dental Impressions of Different Types and Viscosities

**DOI:** 10.3390/ijms231810859

**Published:** 2022-09-17

**Authors:** Joanna Wezgowiec, Anna Paradowska-Stolarz, Andrzej Malysa, Sylwia Orzeszek, Piotr Seweryn, Mieszko Wieckiewicz

**Affiliations:** 1 Department of Experimental Dentistry, Wroclaw Medical University, 50-425 Wroclaw, Poland; 2 Division of Dentofacial Anomalies, Department of Maxillofacial Orthopedics and Orthodontics, Wroclaw Medical University, 50-425 Wroclaw, Poland

**Keywords:** dental materials, ozone, UVC, tensile strength, dimensional stability, hardness

## Abstract

There is an ongoing search for novel disinfection techniques that are not only effective, cheap, and convenient, but that also do not have adverse effects on the properties of dental impressions. We compared the effects of various methods (UVC, gaseous ozone, commercial solution, and spray) on the dimensional change, tensile strength, and hardness of silicone impressions. Moreover, as a secondary aim, we performed a statistical comparison of the properties of nondisinfected addition (Panasil Putty Soft, Panasil monophase Medium, Panasil initial contact Light) and condensation silicones (Zetaplus Putty and Oranwash L), as well as a comparison of materials of various viscosities (putty, medium-bodied, and light-bodied). Our results revealed that addition silicones had higher dimensional stability, tensile strength, and Shore A hardness compared to condensation silicones. Both traditional (immersion and spraying) and alternative methods of disinfection (UVC and ozone) had no significant impact on the tensile properties and dimensional stability of the studied silicones; however, they significantly affected the hardness, particularly of Oranwash L. Our study demonstrated that, similarly to standard liquid disinfectants, both UVC and ozone do not strongly affect the material properties of most silicones. However, before recommendation, their usefulness for each individual material should be thoroughly evaluated.

## 1. Introduction

Dental impressions are negative imprints of oral tissues (teeth, gums, and alveolus). Formation of these impressions is a key stage in the fabrication of dental casts used in prosthetics and orthodontics. Because dental impressions allow the creation of replicas of patients’ teeth and mouth structures, they play an important role in the appropriate diagnosis and precise design of well-fitted oral appliances. Although digital dentistry is fast evolving and displacing conventional methods, dental scans, which are a promising means of taking impressions, still have limitations. One of the issues resolved in modern scanners is the low quality of these scans if the abutment is inclined or the span is too long [1]. Moreover, restorative materials present in the oral cavity may reflect light and cause disturbances in three-dimensional images [2,3,4,5,6]. Therefore, the conventional impression technique is often preferred for obtaining full-arch impressions, which are particularly functional impressions for complete denture fabrication [7].

Materials selected for dental impressions can significantly influence the accuracy and precision of the impression and, consequently, of the final result. Although both rigid (impression plaster and zinc oxide-eugenol) and elastic materials (agar, alginate, polyether, condensation silicone (C-silicone), addition silicone (A-silicone), and polysulfide) have been widely used for creating dental impressions, elastic ones are often preferred. Nowadays, sodium alginate is used as the basic material for taking impressions before the preparation of diagnostic gypsum casts, individual trays, orthodontic appliances, and splints. However, sodium alginate-based impressions are not recommended for more precise applications because they exhibit dimensional instability as they absorb water and swell, and they also constrict due to syneresis [8]. Dental works of the best quality can be achieved using casts made from elastomeric impressions [9]. Silicones are often preferred by dentists as they are characterized by high flexibility and recovery during removal from the oral cavity, as well as the ability to be poured up to 1 week with only slight changes in their dimensional stability, estimated at 0.3% [10].

Apart from good dimensional stability, the ideal impression material should meet other criteria, such as appropriate setting time, flow properties, mechanical strength, accuracy, compatibility with cast materials, safety, ease of manipulation, low cost, and disinfectability. Depending on the application, materials with optimal properties are selected. The analysis of the properties of a dental impression material cannot be limited to the properties of the material itself, in its native form, but must also take into account the impact of time, as well as storage and disinfection conditions, on the material characteristics. Dental impressions must be disinfected in order to limit the risk of cross-contamination and ensure the safety of both patients and dental personnel. Because dental impressions are placed in the oral cavity, where they will be exposed to saliva and blood, and as a result potentially contaminated with pathogens (e.g., streptococci, staphylococci, Escherichia coli, Mycobacterium tuberculosis, hepatitis C virus, and Herpes simplex virus, Candida albicans) [11,12,13], it is important to disinfect them to prevent the transmission of infectious agents between dental offices and laboratories [8,14]. It has been estimated that approximately 70% of the materials transported to dental laboratories are contaminated with microorganisms [15]. Moreover, in the present situation of the SARS-CoV2 pandemic, special attention should be paid to safety concerns as saliva can be a perfect environment for the growth of infectious microorganisms [16].

Currently, there is no “gold standard” method for the disinfection of dental impressions. Immersion is considered to be one of the safest methods to prevent cross-contamination [11]. However, chemical disinfectants may influence the properties of dental impression materials, and consequently, the quality and precision of dental casts and the final prosthetic or orthodontic works [8]. It is known that disinfectants containing oxidizing compounds (e.g., peroxysulfates, sodium hydrochlorite, aldehydes, quaternary ammonium salts, iodine compounds, or organic alcohols) can affect the quality of impression materials, reducing their ability to reproduce the details of the teeth and mucosa. Therefore, other disinfection methods are preferable, and the antimicrobial efficiency of various disinfection procedures and their impact on impression materials must be thoroughly investigated.

Due to the ability to oxidize phospholipids and lipoproteins, ozone may be used for the inactivation of bacteria, viruses, fungi, yeast, and protozoa. Ozone can disrupt the integrity of bacterial cell envelope, damage viral capsid, and prevent virus-to-cell contact [17,18]. Several studies have demonstrated that ozone is a promising agent for surface disinfection, but there is no standard protocol defining both concentration and time of exposure [19]. Some research groups have proposed that ozone can also be used for the disinfection of dental impressions, but this idea is still not widely applied [20]. Moreira Fonseca et al. demonstrated that exposure to ozone caused morphological damage in Streptococcus mutans bacteria due to its ability to induce a significant increase in the production of reactive oxygen species [21]. Apart from the ozonation, exposure to UVC is also considered a promising method of disinfection. Aeran et al. analyzed the effectiveness of UVC radiation in the disinfection of alginate, A-silicone, and polyether impression materials. However, their study revealed only a decrease in the count of bacterial colonies, without investigating the possible adverse effects on material properties [22].

In our previous study, we evaluated the effectiveness of both traditional (commercial spray and solution) and alternative (ultraviolet C (UVC) and gaseous ozone) methods in the disinfection of dental impression materials contaminated with common oral pathogens [23]. All these techniques were found to be effective in reducing bacterial growth on the surface of specimens, but our results suggested the need to further assess them in terms of their influence on the physical and mechanical properties of dental silicones.

In the present study, we aimed to evaluate the effects of disinfection by immersing, spraying, UVC, and ozone on the linear dimensional changes, tensile strength, and hardness of various impression materials (A-silicones and C-silicones of putty-type, medium-bodied, and light-bodied viscosity). In addition to the main research aim, in order to provide more comprehensive insight, as a preliminary step we compared the material properties of different types of silicones without disinfection (addition and condensation silicones), as well as of nondisinfected silicones of various viscosities (putty, medium-bodied, and light-bodied). We tested the following research hypotheses: (1) there is no significant difference in selected material properties between disinfected and nondisinfected dental silicones; and (2) there is no significant difference in the selected material properties between A-silicones and C-silicones as well as between materials of different viscosities.

## 2. Results

### 2.1. Preliminary Comparison of Nondisinfected Materials

The results of the comparison of linear dimensional change, tensile strength, and Shore A hardness between various nondisinfected dental impression materials are presented in Table 1.

Additionally, to draw more general conclusions, a summary of the results of the linear dimensional change test, tensile strength test, and Shore A hardness test performed jointly for each type of silicone (A-silicones and C-silicones) and materials of different viscosity are presented in Table 2 and Table 3, respectively.

The results of the analysis of dimensional changes in the studied impression materials caused by 24-h storage are listed in Table 1, Table 2 and Table 3.

A comparison of nondisinfected controls of each of the studied materials indicated the lowest dimensional changes in Panasil Putty Soft and highest changes in Oranwash L among the studied materials (Table 1). The dimensional changes in A-silicones were significantly lower than those observed in C-silicones (0.2196% vs. 0.5561%, *p* = 0.0082) (Table 2). Furthermore, the dimensional stability of putty and medium-bodied materials was better than that of light-bodied materials (*p* = 0.0142 and *p* = 0.0071, respectively) (Table 3).

The analysis of nondisinfected specimens revealed that tensile strength significantly differed in the impression materials. The value of the parameter was the highest in Panasil monophase Medium (3.607 MPa) and the lowest in Oranwash L (1.159 MPa) (Table 1). In general, A-silicones were characterized by a significantly higher tensile strength than C-silicones (3.043 vs. 1.425 MPa, *p* < 0.0001) (Table 2). Furthermore, in the case of both A-silicones and C-silicones, putty materials had significantly higher tensile strength than light-bodied ones (2.542 vs. 1.748 MPa, *p* = 0.0207) (Table 3). Interestingly, Panasil monophase Medium (material of medium-bodied viscosity) had the highest tensile strength among the studied A-silicones (3.607 MPa) (Table 1).

Similar to the tensile strength test, the results of the analysis of Shore A hardness revealed that the impression materials significantly differed in their hardness. Among the silicones studied, Panasil monophase Medium had the highest Shore A hardness (64.66), and Oranwash L had the lowest value (19.98) (Table 1). In general, when comparing nondisinfected specimens, A-silicones were found to have significantly higher Shore A hardness than C-silicones (59.91 vs. 39.01, *p* < 0.0001) (Table 2). Similar to tensile strength, in the case of both types of silicones, Shore A hardness was significantly higher in putty materials than light-bodied ones (59.55 vs. 37.01, *p* < 0.0001) (Table 3). Among the studied materials, Panasil monophase Medium showed the highest hardness (64.66, *p* < 0.01 for all comparisons) (Table 1).

**Figure 1:**
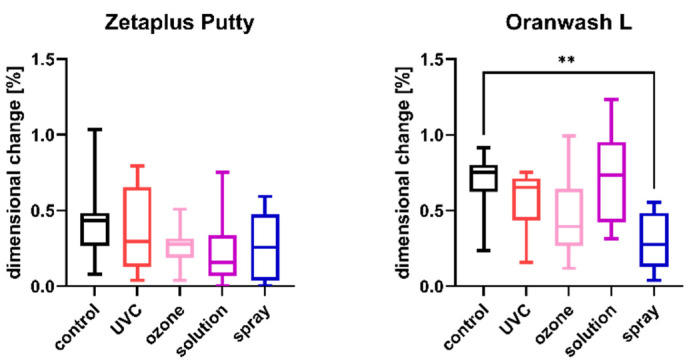
Linear dimensional change in C-silicones (Zetaplus Putty and Oranwash L) after disinfection using various methods; ** *p* < 0.01 for the comparison between the study (disinfected) groups and the control (nondisinfected) group.

**Figure 2:**
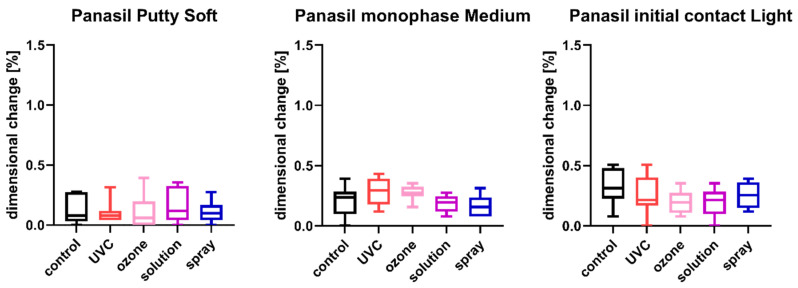
Linear dimensional change in A-silicones (Panasil Putty Soft, Panasil monophase Medium, and Panasil initial contact Light) after disinfection using various methods.

**Figure 3:**
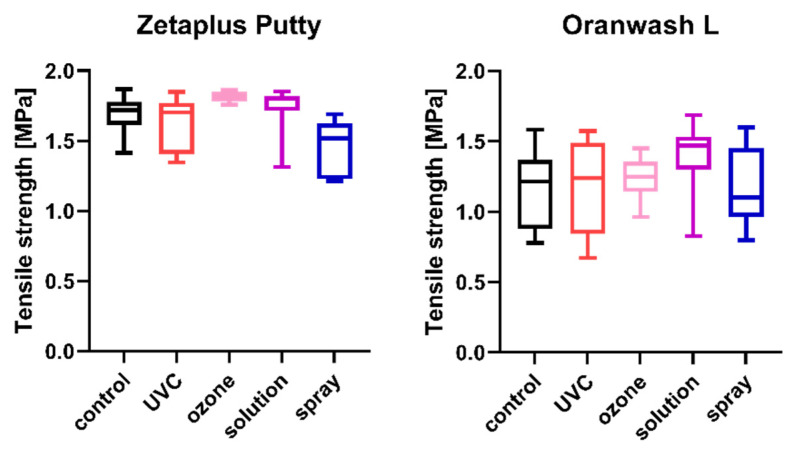
Tensile strength of C-silicones (Zetaplus Putty and Oranwash L) after disinfection using various methods.

**Figure 4:**
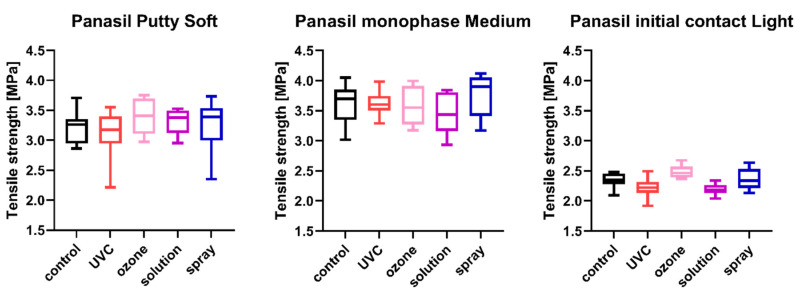
Tensile strength of A-silicones (Panasil Putty Soft, Panasil monophase Medium, and Panasil initial contact Light) after disinfection using various methods.

**Figure 5:**
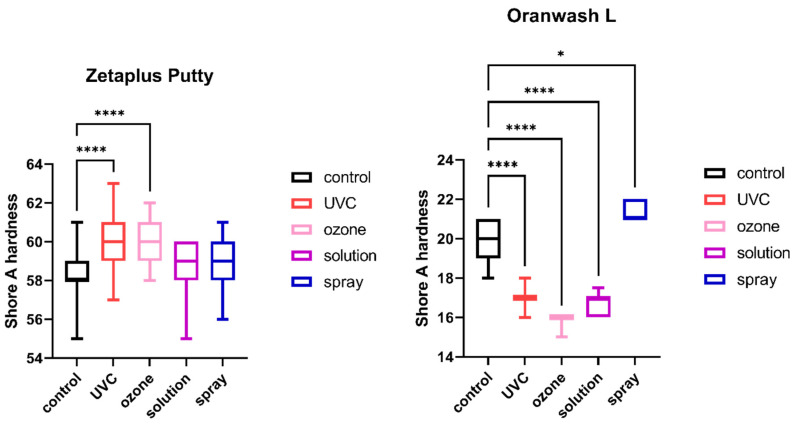
Shore A hardness of C-silicones (Zetaplus Putty and Oranwash L) after disinfection using various methods; * *p* < 0.05 and **** *p* < 0.0001 for the comparison between the study (disinfected) groups and the control (nondisinfected) group.

**Figure 6:**
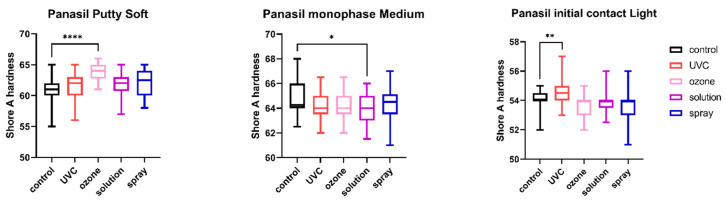
Shore A hardness of A-silicones (Panasil Putty Soft, Panasil monophase Medium, and Panasil initial contact Light) after disinfection using various methods; * *p* < 0.05, ** *p* < 0.01, and **** *p* < 0.0001 for the comparison between the study (disinfected) groups and the control (nondisinfected) group.

The results of the comparison of nondisinfected control and materials disinfected by different techniques are summarized in Figure 1, Figure 2, Figure 3, Figure 4, Figure 5 and Figure 6.

### 2.2. Linear Dimensional Change

The results of the analysis of dimensional changes in the studied impression materials caused by various disinfection techniques are illustrated in Figure 1 (for disinfected C-silicones) and Figure 2 (for disinfected A-silicones).

A comparison of dimensional changes of materials subjected to various disinfection techniques with that of nondisinfected controls revealed that most of the disinfection methods had no significant effect on this parameter of the majority of the studied materials. Only one combination (Oranwash L and spraying with Zeta 7 spray) resulted in a significantly lower dimensional change compared to the nondisinfected control (*p* = 0.0053) (Figure 1). In the case of A-silicones, disinfection did not significantly affect their linear dimensional change in comparison to the nondisinfected control (Figure 2).

### 2.3. Tensile Strength

The tensile strength values measured for each of the studied impression materials are presented in Figure 3 (for disinfected C-silicones) and Figure 4 (for disinfected A-silicones). A comparison of disinfected specimens with nondisinfected controls revealed that disinfection had no effect on the tensile strength of impression materials. Moreover, the tensile strength of studied materials disinfected by different methods did not significantly differ from that of the nondisinfected control (all *p*s > 0.05) (Figure 3 and Figure 4).

### 2.4. Shore A Hardness

The results of the analysis of Shore A hardness of each of the studied impression materials are presented in Figure 5 (for disinfected C-silicones) and Figure 6 (for disinfected A-silicones). Disinfection had a significant impact on hardness. This effect was particularly evident for C-silicones (significant difference from nondisinfected specimens at *p* < 0.05 and Zetaplus disinfected by UVC and ozone disinfection and Oranwash L disinfected by different disinfection methods) (Figure 5). In addition, the hardness of putty-type materials (both Zetaplus and Panasil Putty Soft) increased after disinfection, while in the case of light- and medium-bodied silicones it was reduced by most of the studied disinfection methods (Figure 5 and Figure 6).

## 3. Discussion

Despite high awareness of the importance of disinfecting dental impressions, which is a routine procedure in dental offices and laboratories, this practice is often neglected, particularly in developing countries [15]. Research on this topic is highly desirable, provided that it is reliable, because the introduction of cheaper and more convenient methods could improve the accessibility of disinfection procedures and increase their frequency. A systematic review and meta-analysis by AlZain revealed several discrepancies between the findings of studies undertaken to determine the effect of different disinfection methods on the properties of various impression materials, indicating that such research needs to be better designed and standardized [24]. Therefore, we performed this comprehensive study on dental silicones of various types and of different viscosities, subjecting them to both traditional and alternative methods of disinfection. We chose UVC radiation and gaseous ozone as nonstandard, low-cost, and convenient methods.

Our previous study showed that ozone disinfection (for 10 min at 15 ppm concentration and 800 mg/h flow rate) significantly reduced the growth of pathogens (Pseudomonas aeruginosa, S. aureus, and C. albicans) recommended by ISO standards for the evaluation of the bactericidal [25] and fungicidal or yeasticidal [26] activity of chemical disinfectants. Our research also confirmed the efficacy of exposure to UVC for 40 min in the disinfection of A-silicones and C-silicones [23].

Before recommendation, any newly discovered disinfection method must be evaluated for antimicrobial effectiveness as well as the potential influence on the material properties of dental impressions. For this purpose, we continued our study to demonstrate the efficacy and safety of UVC radiation and gaseous ozone in the disinfection of various impression silicones [23]. As their effects were similar to commercial solution and spray, in the present study we evaluated their influence on the physical properties of selected elastomeric materials.

One of the most important properties of dental impressions is their dimensional stability, which allows the appropriate adaptation of prosthetic or orthodontic appliances that is essential for their long-term clinical success. This parameter of impression materials may be strongly affected by disinfection, and thus most studies have focused on the dimensional stability and surface quality of impression materials after disinfecting them with various liquid disinfectants [11,27,28,29,30]. Özdemir and Pekince found that the dimensional changes of materials (polyether, hydrocolloid, C-silicone, and A-silicone) disinfected with 1% sodium hypochlorite or an aldehyde-free disinfectant solution (Zeta 7 spray) differed depending on the type of impression material, storage time, and disinfectant solution used. Nevertheless, the values were within the clinically acceptable level specified by the American Dental Association (ADA) (<0.5%) [28]. A comparison of the dimensional stability of various materials (polysulfide, A-silicone, C-silicone, and polyether) after immersion in a 5.25% sodium hypochlorite solution and in a 2% glutaraldehyde solution showed a significant difference among the elastomers used but no change in the dimensional stability of elastomers after disinfection in comparison to the control group [14]. Similar results were revealed by our study, indicating that the studied methods of disinfection had no negative effect on the dimensional changes of any studied material. On the other hand, a comparison of various materials before disinfection showed that the dimensional stability of A-silicones was significantly higher than that of C-silicones. Such differences were also confirmed by the other groups, demonstrating that due to the release of setting reaction by-products, C-silicones showed higher dimensional changes than A-silicones [31,32,33]. Furthermore, our results showed minor dimensional changes in non-disinfected putty and medium-bodied materials compared with light-bodied silicones, which may be explained by the lower filler content of the light-bodied materials. Khinnavar et al. also confirmed a similar dependence of dimensional stability on the viscosity of impression materials [34].

To reduce dimensional changes of dental elastomers, various alternatives to chemical sterilization have been looked for, such as steam autoclaving, microwave disinfection, or UVC radiation [35,36]. Samra et al. reported that exposure to UVC radiation for 3 min did not significantly influence the dimensional stability of alginates and A-silicones in comparison to nondisinfected controls. However, their study did not evaluate the antimicrobial effectiveness of such a short period of disinfection [36]. Another study also showed that UVC disinfection for a short time (10 min) had no effect on the dimensional stability of both putty and light-bodied addition-type silicone (Reprosil) [37]. Furthermore, it was shown that UVC radiation for 20 min did not influence the dimensions of the impression taken using heavy body elastomeric material followed by the wash impression based on light-body impression material (Aquasil) [38]. In our study, we applied UVC for 40 min, in accordance with the recommendations of the UVC lamp manufacturer and a previous paper proving the effectiveness of this disinfection period [23].

Poulis et al. qualitatively evaluated the effect of ozone disinfection on the surface of impression materials [39]. In the first study, the authors observed that ozone exposure caused significant eradication of Gram (+) (S. Aureus) and Gram (–) (Klebsiella pneumoniae) bacteria from the surface of light-body addition-cured silicone (Aquasil Ultra LV-Regular Set) [40]. In the next study, they observed similar wavy-wrinkling structures on the surface of specimens treated with either a 0.3% benzalkonium chloride solution or ozone. These effects could have resulted from the surface oxidation of vinyl polysiloxane by ozone or liquid chemicals, due to the fracture of methyl groups present on the surface of the polymer chain, and the subsequent formation of a stiff silicon dioxide film. The authors concluded that qualitative scanning electron microscopic analysis should be followed by a quantitative assessment to obtain a more detailed insight [39]. Our research showed no significant effect of ozone disinfection on the dimensional change of impression materials.

Another important property of dental impression materials is their tensile strength, which refers to the ability to withstand tensile forces that are applied on the impression material during its removal from the mouth to separate it from the teeth and surrounding tissues. In other words, tensile strength is the maximum stress a material can withstand under uniaxial traction before it ruptures [41]. This property is considered particularly important as the material is exposed to interproximal spaces, sharp line angles, and gingival crevices [10]. A comparison of two types of vinylpolysiloxane impression materials (autoclavable Affinis and nonautoclavable Aquasil) revealed the differences in tear strength and tensile strength between them; however, their properties were not changed upon disinfection [10]. Kotha et al. compared five types of addition-reaction polyvinylsiloxane putty impression materials and concluded that chemical disinfection and autoclave sterilization did not significantly affect their tensile strength, while microwave sterilization reduced this value to a great extent [42]. However, there is a need for a comparative study of the tensile strength of disinfected and nondisinfected elastomers of various types and viscosities, as well as a study of the effects of UVC and ozone disinfection. Our results showed that the tensile properties of both C-silicones and A-silicones of various viscosities were not significantly influenced by standard chemical disinfectants and UVC or gaseous ozone, but these materials themselves significantly differed in their ability to withstand tensile forces, which is in line with the main conclusion of Gupta et al. [10]. Furthermore, our study confirmed the findings of Meincke et al. that materials with lower filler content have lower tensile strength, while more viscous materials can withstand higher tensile forces due to the increased amount of filler [41].

According to our study, Shore A hardness is the physical property of the impression materials most affected by disinfection. Among the studied materials, light-bodied C-silicone (Oranwash L) was characterized by the lowest Shore A hardness, even without disinfection. Changes in hardness could possibly affect the convenience of working with this material and thus the accuracy of the final casts. Hence, the applicability of various methods of disinfection should be thoroughly evaluated for each material, especially condensation-curing materials of lower viscosity. Interestingly, despite the importance of this parameter, changes in the hardness of the impression materials upon disinfection have not been thoroughly investigated so far. Goiato et al. explained that one reason for the significantly lower hardness value observed in the material disinfected by immersion is absorption of the disinfection solution, and the degree of absorption is dependent on the filler material and the low level of adhesion between silicone polymers [43]. Overall, two factors were proposed as a cause of the decrease of hardness value: scission of polymer’s chain that may increase the freedom of molecules’ movement, and absorption of the water, which may act as an additional plasticizer enhancing material resiliency. Conversely, oxygen crosslinking and leaching of plasticizers may be causes of the increased hardness by reduction in the molecules’ movements and reduction of material elasticity [44]. As little is known about the possible mechanisms of action of various methods of disinfection on the material hardness and surface quality, this is an interesting research direction still waiting for an in-depth investigation.

In terms of clinical application of impression materials, all the parameters studied are of great importance. Dimensional stability, tensile strength, and hardness strongly influence the precision of dental restoration, since tensile forces act on the impression during its removal from the patient’s mouth and could cause deformations, while good dimensional stability guarantees proper accuracy of restoration despite a time delay between the taking of the impression and the pouring of the gypsum cast. Hardness of the material is the characteristic that is important from the point of view of easy manipulation of the impression after setting, as the reduction of the hardness of the material could pose the risk of damage of the impression during manipulation with the modeling tools. For these reasons, the material’s characteristics should be not changed upon disinfection.

Our study revealed the differences between the analyzed dental impression materials, even without disinfection. The linear dimensional changes of A-silicones were significantly lower than those of C-silicones, while the tensile strength and Shore A hardness of the A-silicones were significantly higher than the C-silicones. Additionally, the dimensional stability, tensile strength, and Shore A hardness of putty-type and medium-bodied materials were better compared to light-bodied material. Most of the disinfection methods applied did not affect the dimensional stability and tensile strength of a majority of the studied materials. On the other hand, disinfection significantly affected the hardness of the materials, especially the C-silicones. For this reason, based on our results, only disinfection by spraying could be recommended for Oranwash L, since all other methods significantly reduced the hardness of this material. For the other silicones, all methods could be applied, as they did not worsen the material properties studied.

Future research on impression materials should analyze the influence of disinfection on their other properties, since it has been shown that some of these (e.g., surface roughness) can be significantly affected, particularly by chemical disinfectants [45,46]. Moreover, although our study applied a carefully designed methodology, simulating most of the oral conditions such as temperature and moisture, there is a need for more studies to confirm the lack of adverse effects of the proposed methods of disinfection. Clinical investigations would provide valuable information on the changes brought by disinfection in impression materials, as a complex geometry of the oral cavity may affect these dependencies.

## 4. Materials and Methods

**Figure 7:**
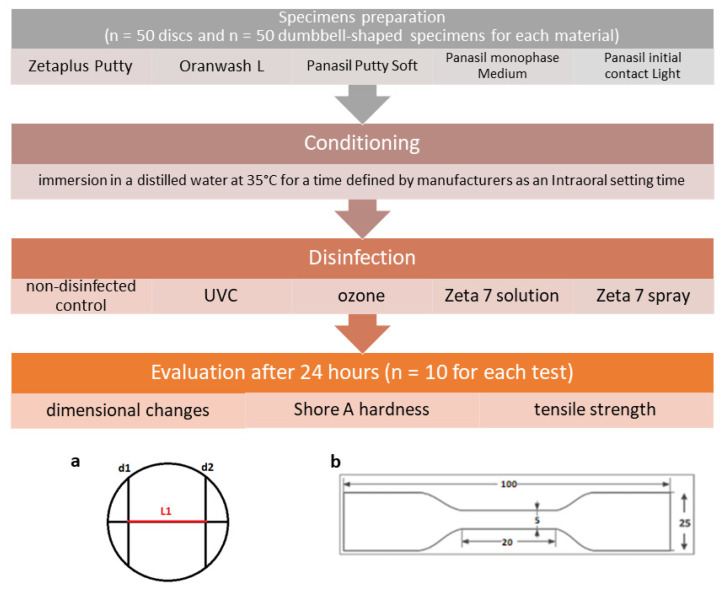
Study design with a schematic representation of (**a**) the test block used for the measurement of dimensional change in accordance with the ISO 4823:2015 standard and (**b**) the dumbbell-shaped test specimen used for the measurement of tensile strength in accordance with the ISO 37:2017(E) standard.

The general design of the study is depicted in Figure 7.

### 4.1. Materials

The study examined five dental silicones: three condensation-curing silicones (Zetaplus Putty, Oranwash L, and Panasil Putty Soft) and two addition-curing silicones (Panasil monophase Medium and Panasil initial contact Light). These materials had different degrees of viscosity (Table 4).

### 4.2. Preparation of Specimens

The materials were processed according to the manufacturer’s recommendations. Zetaplus Putty and Panasil Putty Soft were mixed using fingertips, Oranwash L using a spatula and clean mixing block, Panasil monophase Medium and Panasil initial contact Light using a dispensing gun with mixing tips.

Samples were prepared following ISO 37:2017(E) [47] (specimens for the determination of tensile strength) or ISO 4823:2015 [48] (specimens for the determination of dimensional stability and Shore A hardness) standards. Stainless steel dies (Slusarstwo-Tokarstwo Paweł Sitkowski, Wroclaw, Poland) and a self-made die based on dental stone (GC Fujirock EP Premium Line Super Hard Plaster, GC Corporation, Tokyo, Japan) were used for the preparation of samples.

After mixing, the materials were transferred to a proper die, covered and pressed with a glass plate, and conditioned in an incubator (CLN 15 Smart, POL-EKO-APARATURA, Wodzislaw Slaski, Poland). Then, the materials were immersed in distilled water at 35 °C for a duration specified by the manufacturers as an intraoral setting time/time in the oral cavity (Table 4). After conditioning, the specimens prepared for the tensile strength test were left for 3 h before they were cut with a cutter (Slusarstwo-Tokarstwo Paweł Sitkowski, Wroclaw, Poland) and disinfected. The specimens prepared for dimensional stability and hardness tests were removed from the die, rinsed with tap water, and disinfected using different methods selected for this study.

### 4.3. Disinfection

The prepared specimens were subjected to various methods of disinfection. Their parameters are detailed in Table 5. For each type of the studied materials, specimens not subjected to any method of disinfection were used as nondisinfected controls.

After disinfection, the specimens were kept in closed boxes for 24 h at room temperature, and then their properties were evaluated. All tests were carried out at the standard laboratory temperature of 23 ± 2 °C and humidity of 50 ± 10%.

### 4.4. Linear Dimensional Change Test

The effect of different disinfection techniques on the dimensional stability of the studied dental silicones was evaluated in accordance with the ISO 4823:2015 standard [48]. For this purpose, the distance between lines *d_1_* and *d_2_* along line *c* was measured on a test block and on specimens using a Magnusson digital caliper (150 mm) (Limit, Wroclaw, Poland). The dimensional changes, Δ*L* [%], were calculated using the following equation:

Δ*L* = 100 × (*L*_1_ − *L*_2_)/*L*_1_ (1)

where

*L*_1_ is the distance measured between lines *d_1_* and *d_2_* in the test block [mm]; and
*L*_2_ is the distance measured between lines *d_1_* and *d_2_* in the impression material specimen [mm].

### 4.5. Tensile Strength Test

The effect of different disinfection techniques on the tensile strength of the studied dental silicones was evaluated in accordance with the ISO 37:2017(E) standard [47]. Type 1A dumbbell test pieces were selected for evaluation as they have advantages over types 1 and 2 due to better repeatability and particularly lower number of breaks outside the test length. Specimens with a standard thickness of 2.0 mm were obtained using a specially fabricated cast die based on dental stone (GC Fujirock EP Premium Line Super Hard Plaster, GC Corporation, Tokyo, Japan). Dumbbells of proper dimensions were prepared using the appropriate steel cutter. The thickness and width of specimens were measured three times using a digital caliper (Limit, Wroclaw, Poland), and the cross-sectional area was calculated. Then, using the Universal Testing Machine (model Z10-X700, AML Instruments, Lincoln, UK), the tensile test was performed with the nominal traverse rate of moving grip (500 mm/min). Any test piece that broke outside the test length was discarded, and a repeat test was conducted on another test piece.

Tensile strength, *TS* [MPa], was calculated using the following equation:

*TS* = *F_m_*/(*W* × *t*) (2)

where

*F_m_* is the maximum force [N];
*t* is the thickness of the test piece over the test length [mm]; and
*W* is the width of the test piece over the test length [mm].

### 4.6. Hardness Test

The Shore A hardness of the specimens was evaluated according to the PN-EN ISO 868:2005 standard [49]. The measurements were made at five different points using the Sauter HBA 100-1 Shore-Durometer (KERN & SOHN GmbH, Balingen, Germany) attached to the Sauter Durometer Test Stand TI-A0 (KERN & SOHN GmbH).

### 4.7. Statistical Analysis

The obtained results were analyzed using GraphPad Prism 9.1.2. software (GraphPad Software, San Diego, CA, USA). All measurements were carried out for a minimum of 10 samples (n ≥ 10 for each group). The results were expressed as box pots displaying the five-number summary of a set of data (minimum, first quartile, median, third quartile, and maximum). Differences between the studied methods of disinfection and nondisinfected controls were tested using parametric one-way analysis of variance (ANOVA) with post hoc Tukey’s Honest Significant Difference test or the nonparametric Kruskal–Wallis ANOVA with post hoc Dunn’s test. Differences between the groups were considered statistically significant at *p* < 0.05.

## 5. Conclusions

Our general conclusion is that both the main and additional hypotheses of the study should be rejected. The results showed that: (1) A-silicones had better dimensional stability, tensile strength, and Shore A hardness than C-silicones; and (2) both traditional (immersing and spraying) and alternative (UVC and ozone) disinfection methods did not significantly influence the tensile properties and dimensional stability of the studied dental silicones; however, disinfection significantly affected their hardness, particularly that of light-bodied C-silicone (Oranwash L).

Based on our results, it can be concluded that both UVC radiation and gaseous ozone may be promising alternative methods for disinfecting dental impressions, especially A-silicones and putty-type C-silicones. Because these disinfection techniques may have a negative influence on the hardness of impressions, their usefulness for light-bodied silicones should be assessed for each individual material.

## Figures and Tables

**Table 1:** Mean (SD) of linear dimensional change, tensile strength, and Shore A hardness for nondisinfected controls of various impression materials; different superscript letters indicate the statistical significance (*p* < 0.05) of differences between the studied groups.

Material	Linear Dimensional Change [%]	Tensile Strength [MPa]	Shore A Hardness
Zetaplus Putty	0.4190 (0.2626) ^ab^	1.691 (0.1345) ^d^	58.08 (1.383) ^c^
Oranwash L	0.6933 (0.1977) ^a^	1.159 (0.2901) ^e^	19.98 (0.7690) ^e^
Panasil Putty Soft	0.1179 (0.1126) ^c^	3.196 (0.2547) ^b^	61.02 (2.025) ^b^
Panasil monophase Medium	0.2039 (0.1236) ^bc^	3.607 (0.3273) ^a^	64.66 (1.201) ^a^
Panasil initial contact Light	0.3368 (0.1487) ^ac^	2.337 (0.1231) ^c^	54.04 (0.6987) ^d^

**Table 2:** Mean (SD) of linear dimensional change, tensile strength, and Shore A hardness for C-silicones and A-silicones, and the statistical significance (*p*) of differences between these two groups.

Type of Material	Linear Dimensional Change [%]	Tensile Strength [MPa]	Shore A Hardness
C-silicones	0.5561 (0.2664)	1.425 (0.3506)	39.03 (19.18)
A-silicones	0.2196 (0.1547)	3.043 (0.5660)	59.91 (4.640)
Significance	*p* = 0.0082	*p* < 0.0001	*p* < 0.0001

**Table 3:** Mean (SD) of linear dimensional change, tensile strength, and Shore A hardness for the materials of different types of viscosity; different superscript letters indicate the statistical significance (*p* < 0.05) of differences between the studied groups.

Type of Viscosity of Material	Linear Dimensional Change [%]	Tensile Strength [MPa]	Shore A Hardness
Putty	0.2684 (0.2501) ^b^	2.542 (0.7904) ^b^	59.55 (2.271) ^b^
Medium-bodied	0.2039 (0.1236) ^b^	3.607 (0.3273) ^a^	64.66 (1.201) ^a^
Light-bodied	0.5151 (0.2499) ^a^	1.748 (0.6420) ^c^	37.01 (17.13) ^c^

**Table 4:** Description of dental impression materials used in the study.

Type of Material	Viscosity	Name	Manufacturer	Mixing Technique	Intraoral Setting Time at 35 °C
C-silicone (condensation polysiloxane)	Putty	Zetaplus Putty	Zhermack (Badia Polesine, Italy)	Manual (hand mix)	3 min 15 s
	Light-bodied	Oranwash L		Manual (with spatula)	3 min 30 s
A-silicone (vinyl polysiloxane)	Putty	Panasil Putty Soft	Kettenbach (Eschenburg, Germany)	Manual (hand mix)	2 min
	Medium-bodied	Panasil monophase Medium		Dispensing gun with mixing tip	2 min
	Light-bodied	Panasil initial contact Light		Dispensing gun with mixing tip	2 min 30 s

**Table 5:** Parameters of the disinfection methods applied in this study.

Method	Material or Equipment	Description
UVC	UV-C Blue (Activeshop, Wroclaw, Poland)	Irradiation for 40 min at 254 nm
Ozone	Ozox Professional G168 (MediaSklep24, Bojszowy, Poland)	Putting in an 8-L box with 15 ppm ozone concentration, and ozonation for 10 min at an ozone flow rate of 800 mg/h
Solution	Zeta 7 Solution (Zhermack, Badia Polesine, Italy); active ingredients: quaternary ammonium salts, phenoxyethanol	Immersion for 10 min in 100-time diluted solution and rinsing with distilled water
Spray	Zeta 7 spray (Zhermack, Badia Polesine, Italy); active ingredients: alcohols	Spraying all surfaces of the specimen and allowing to dry

## Data Availability

The datasets generated and/or analyzed during the study are available from the corresponding author on reasonable request.
